# Supplementary material for: TMPRSS2 Is Essential for SARS-CoV-2 Beta and Omicron Infection
Source: Viruses. 2023 Jan 18;15(2):271. doi: 10.3390/v15020271 (PMC9961888; doi:10.3390/v15020271)
Supplement: Supplementary file 1 [file viruses-15-00271-s001.zip › viruses-2120570-supplementary.pdf]

viruses

Article

TMPRSS2 is essential for SARS-CoV-2 Beta and Omicron infection

Kristin Metzdorf [1,11], Henning Jacobsen [1,11], Marina C. Greweling-Pils [2], Markus Hoffmann [3,5], Tatjana Lüddecke [1], Felicitas Miller [2], Lars Melcher [4], Amy M. Kempf [3,5], Inga Nehlmeier [3], Dunja Bruder [4,6], Marek Widera [7], Sandra Ciesek [7,8,9], Stefan Pöhlmann [3,5] and Luka Čičin-Šain [1,10,*]

Supplementary Materials:

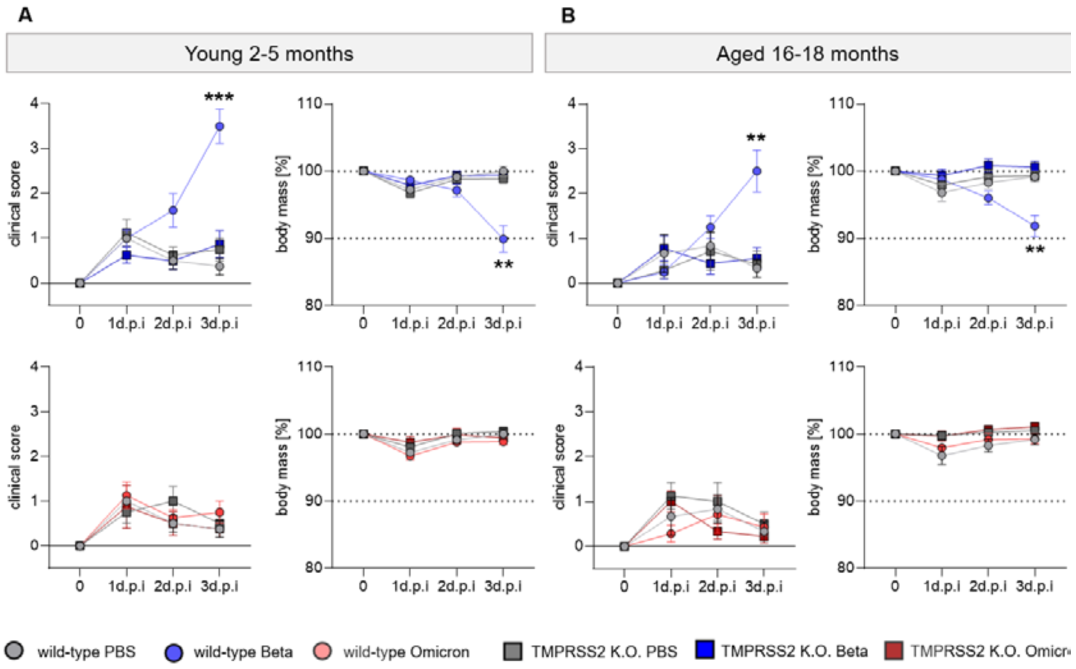

Supplementary Figure S1: SARS-CoV-2 Beta and Omicron pathogenesis in young and aged mice. Wild-type and TMPRSS2 K.O. mice were intra-nasally infected with $1x10^4$ PFU of SARS-CoV-2 Beta or Omicron. Intra-nasal PBS-application was used as treatment control. **(A)** Young 2-5 months old mice show significantly altered clinical scores and body mass [%] for Beta infected wild-type mice at 3 dpi. Omicron infected young mice show no difference. Animal numbers: each group n=8 **(B)** Aged 16-18 months old show equal changes in clinical scores and body mass. Significant increase in clinical score and reduction in body mass can be displayed for Beta infected wild-type mice. Omicron infection results in no changes in pathogenesis in aged mice. Animal numbers wt mice: PBS n=8; Beta n=9; Omicron n=8; Animal numbers TMPRSS2 K.O. mice: PBS n=7; Beta n=9; Omicron n=9 (A, B) Statistical significance for sequential body mass and clinical scores was calculated using Two-Way ANOVA with Geisser-Greenhouse correction and multiple comparison and correction for multiple comparisons following Tukey and are shown as mean ±SEM (**p <0.01, ***p < 0.001).

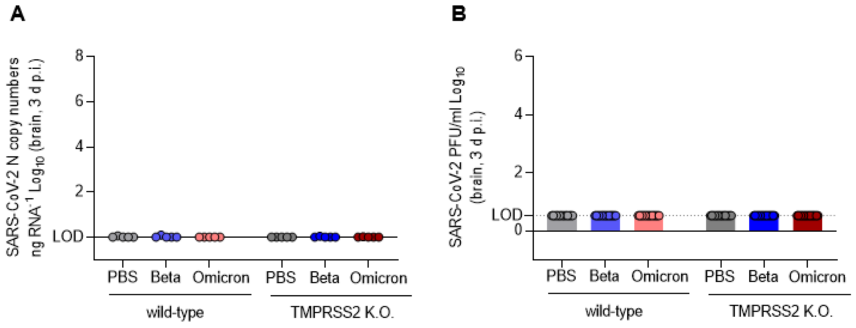

Supplementary Figure S2: Viral load and infectious-virus titers after infection with SARS-CoV-2 Beta and Omicron in brain tissue **(A)** SARS-CoV-2 N gene copy numbers as copy number/ng RNA Log10 at 3 dpi with SARS-CoV-2 Beta (blue) and Omicron (red) in brain homogenates. **(B)** SARS-CoV-2 Beta and Omicron infectious-virus titer as PFU/ml Log10 at 3 dpi in brain homogenates. Dotted lines indicate the limit of detection (LOD) for infectious-virus titers

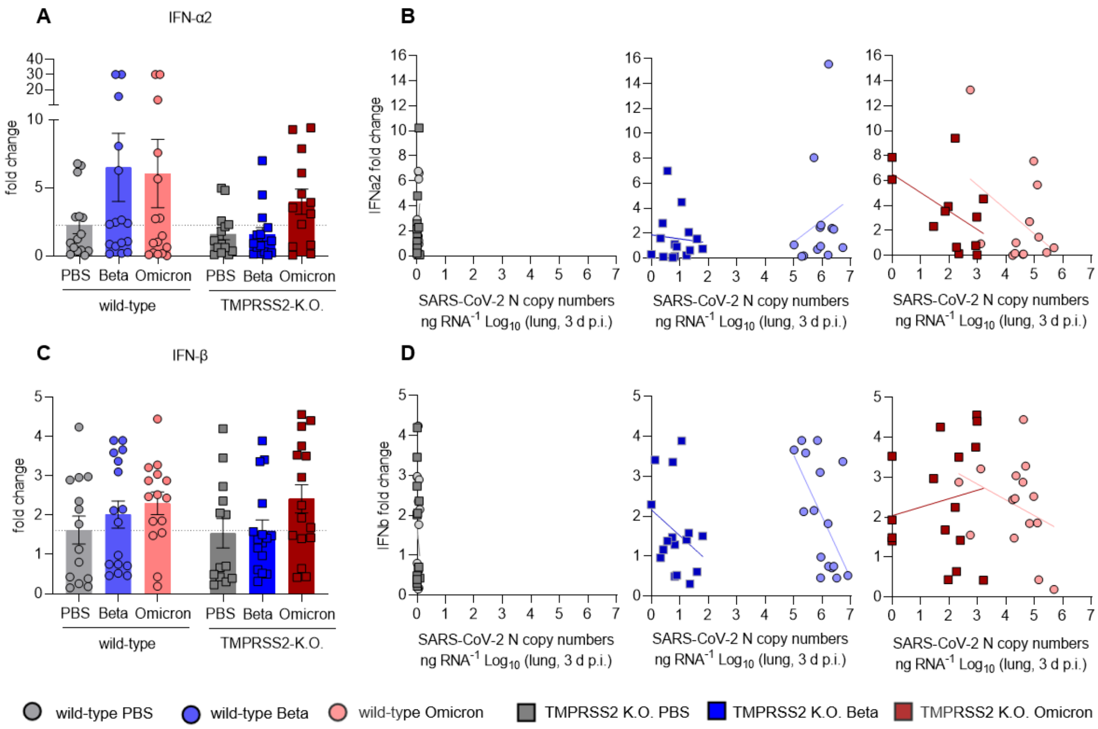

Supplementary Figure S3: TMPRSS2 affects the Type I interferon response in SARS-CoV-2 infected mice. mRNA expression in isolated lung tissue of **(A)** IFNα2 and **(B)** IFNβ was detected in wild-type and TMPRSS2 K.O. mice intra-nasally infected with 1x10[4] PFU of SARS-CoV-2 Beta or Omicron and PBS respectively. The fold change was depicted as mean values ± SEM **(B, D)** Fold change of Type I interferon **(B)** IFNα2 and **(D)** IFNβ correlated to the SARS-CoV-2 N gene copy numbers as copy number/ng RNA Log10 at 3 dpi with PBS (left) SARS-CoV-2 Beta (middle) and Omicron (right) comparing wild-type and TMPRSS2 K.O.
